# Assessment of Alzheimer’s Disease Based on Texture Analysis of the Entorhinal Cortex

**DOI:** 10.3389/fnagi.2020.00176

**Published:** 2020-07-02

**Authors:** Stephanos Leandrou, Demetris Lamnisos, Ioannis Mamais, Panicos A. Kyriacou, Constantinos S. Pattichis, for the Alzheimer’s Disease and Neuroimaging Initiative

**Affiliations:** ^1^School of Science, European University Cyprus, Nicosia, Cyprus; ^2^School of Mathematical Sciences, Computer Science and Engineering, City, University of London, London, United Kingdom; ^3^Department of Computer Science, University of Cyprus, Nicosia, Cyprus; ^4^Research Centre on Interactive Media, Smart Systems and Emerging Technologies (RISE CoE), Nicosia, Cyprus

**Keywords:** Alzheimer’s disease, mild cognitive impairment, entorhinal cortex, magnetic resonance imaging, texture

## Abstract

Alzheimer’s disease (AD) brain magnetic resonance imaging (MRI) biomarkers based on larger-scale tissue neurodegeneration changes, such as atrophy, are currently widely used. Texture analysis evaluates the statistical properties of the tissue image quantitatively; therefore, it could detect smaller-scale changes of neurodegeneration. Entorhinal cortex is the first region affected, and no study has investigated texture analysis on this region before. This study aims to differentiate AD patients from Normal Control (NC) and Mild Cognitive Impairment (MCI) subjects using entorhinal cortex texture features. Furthermore, it was evaluated whether texture has association to MCI beyond that of volume, to evaluate if atrophy development may precede. Texture features were extracted from 194 NC, 200 MCI, 84 MCI who converted to AD (MCIc), and 130 AD subjects. Receiving operating characteristic curves determined the performance of the various features in discriminating the groups, and a predictive model was used to predict conversion of MCIc subjects to AD. An area under the curve (AUC) of 0.872, 0.710, 0.730, and 0.764 was seen between NC vs. AD, NC vs. MCI, MCI vs. MCIc, and MCI vs. AD subjects, respectively. Including entorhinal cortex volume improved the AUCs to 0.914, 0.740, 0.756, and 0.780, respectively. For the disease prediction, binary logistic regression was applied on five randomly selected test groups and achieved on average AUC’s of 0.760 and 0.764 on the training and validation cohorts, respectively. Entorhinal cortex texture features were significantly different between the four groups and in many cases provided better results compared to other methods such as volumetry.

## Introduction

Alzheimer’s disease (AD) represents the first disease in the top 10 disease leading cause of death in the United States (US), and it cannot be prevented, slowed, or cured. It represents the most common form of dementia, which affects memory, language, and other cognitive skills and eventually leads to an inability of everyday activities. Despite continuous advances in exploring the nature of AD, according to the World Health Organization (WHO), there are 47 million patients worldwide, and by 2030, this number is projected to increase to 75 million.

There are still many unresolved issues regarding the pathophysiology of this highly heterogeneous disease in terms of diagnosis and follow-up. In an attempt to understand better the disease, studies have been examining the neuropathology and cognitive impairment in animal models such as transgenic mice (see Kitazawa et al., 2012; Saito et al., 2014 for a review). However, it seems that AD is a uniquely human disease. With no doubt, animal models offer the advantage of *in vivo* testing, which in humans is available only post-mortem; however, they lack main disease pathological features such as neuronal loss or neurofibrillary tangle (NFT) development, which is the neuropathological hallmark of AD (Drummond and Wisniewski, 2017). The diagnosis of the disease still remains probable and only post-mortem biopsy can confirm AD as it reveals deposits of amyloid-β (Aβ) plaque deposition and tau protein (NFTs) in the brain tissue (Braak and Braak, 1997a). However, due to brain inaccessibility, the diagnosis is based on other factors such as medical and family history, input from the family members regarding the behavior of the subject, blood test, imaging, and cognitive tests. Cognitive tests such as the Mini Mental State Examination (MMSE) (Folstein et al., 1975) and the Clinical Dementia Rating (CDR) (Morris, 1993) represent two of the most commonly used tests in the assessment of AD, and they evaluate memory and language abilities. However, an important limitation of clinical and cognitive assessment is the diagnosis of probable AD after structural changes have occurred within the brain (Braak and Braak, 1991, 1996; Morris et al., 1996). Jack et al. (2004) showed that brain atrophy on structural magnetic resonance imaging (MRI) was detected more consistently than decline on cognitive scores in patients with AD. Furthermore, when cognitive assessment is not used in combination with other biomarkers, the former suffers from low sensitivity (Dukart et al., 2016).

Indeed, decades before the first clinical symptoms become apparent, there is an inevitable progression of atrophy, which initially affects the medial temporal lobe (MTL) (Scahill et al., 2002; Petrella et al., 2003; Jack et al., 2004). MTL is highly associated with memory, and as the neurons get affected, cognitive and functional deficits start to appear. Post-mortem studies (Braak and Braak, 1997b; Kordower et al., 2001) have implicated entorhinal cortex as an early site of involvement in AD followed by the hippocampus, the amygdala, and the parahippocampal gyrus (Dickerson et al., 2001; Killiany et al., 2002; Squire et al., 2004).

Mild cognitive impairment (MCI) represents the transitional stage between normal aging and AD, and it cannot be easily identified by cognitive tests. MCI subjects may have decreased memory function beyond the normal level based on a given person’s age and education; however, they do not fulfill the criteria for dementia, as their cognitive function is comparable to Normal Control (NC) subjects. Most of these subjects will remain stable even after 10 years of follow-up (Mitchell and Shiri-Feshki, 2009), and only ∼15% will progress to AD (Farias et al., 2009).

Therefore, due to cognitive assessment limitations especially for MCI subjects, the research community has been actively searching for diagnostic imaging biomarkers especially the ones derived from quantitative T1-weighted MRI. The importance of structural MRI in the assessment of AD was underlined by its inclusion in the new diagnostic criteria (Dubois et al., 2007) along with temporoparietal hypometabolism as seen in positron emission tomography (PET) (Mosconi et al., 2008), positivity on amyloid imaging as seen in PET (Johnson et al., 2013), and abnormal neuronal cerebrospinal fluid (CSF) biomarkers (tau and/or Aβ) (Brier et al., 2016; Bjerke and Engelborghs, 2018; Blennow and Zetterberg, 2018). However, PET studies are not easily accessible, due to several factors such as cost, radiopharmaceutical limitations (availability, targeting amyloid or tau proteins), and exposure to ionizing radiation. On the other hand, structural MRI has no ionizing radiation; thus, it is preferable in longitudinal investigations, and it provides high-resolution images. However, radiologists cannot perceive subtle changes of neurodegeneration, especially in the early stages of the disease by the naked eye observation. Even if they could, without any quantitative measurements, it would be impossible to predict the patient’s progress.

Therefore, research on quantitative MRI-derived biomarkers of AD is an active research area, which can reveal the cerebral atrophy and they are used as a biomarker in the assessment of AD. In the literature, several MRI quantitative methods are described for use in the assessment of AD. Such methods are: (i) voxel-based morphometry (VBM), which describes global changes or atrophy of the deep cerebral structures (ii) volume analysis (iii) thickness analysis (iv) shape, and (v) texture analysis. Volumetry remains the most popular technique to assess AD especially in the area of MTL (Jack et al., 1999; Killiany et al., 2000; Dickerson et al., 2001; Pennanen et al., 2004). Apart from the MTL, other studies chose to assess the whole brain (Misra et al., 2009; Plant et al., 2010), although the cortex is affected in later stage (Braak and Braak, 1991). Multimethod studies (Cuingnet et al., 2011; Wolz et al., 2011; Da et al., 2014) combined biomarkers for a better understanding of the disease pathophysiology and heterogeneity.

The accumulation of NFTs and Aβ plaques is present prior to atrophy, and these plaques could affect image intensity structure and distribution. Texture analysis statistical properties of the image intensities might represent changes in MRI image pixel intensity due to NFTs and Aβ plaques. Furthermore, MRI biomarkers based on texture might be able to detect earlier stages of AD than biomarkers that use larger-scale changes, such as thickness or atrophy. The establishment of such biomarkers will allow the identification of individuals with MCI at an earlier stage. This might lead to a better management of the MCI group targeting in slower progression or even prevention to conversion to AD.

Although hippocampus represents the most established region of interest (ROI) used in the assessment of AD, the earlier involvement of the entorhinal cortex was proved by many studies (Gómez-Isla et al., 1997; Juottonen et al., 1999; Galton et al., 2001; Killiany et al., 2002; Busatto et al., 2003; deToledo-Morrell et al., 2004; Tapiola et al., 2008). In two comprehensive reviews (Zhou et al., 2016; Leandrou et al., 2018), the authors concluded that structural changes in the early stages of the disease are more pronounced in the entorhinal cortex. Table 1 tabulates studies that used entorhinal cortex in the assessment of AD. Furthermore, for the disease prediction, entorhinal cortex provided better predictive accuracies compared to hippocampus. Although volumetry represents the most commonly used method to date, there is lack of research in the assessment of AD using texture analysis. The study of Sørensen et al. (2015), found that hippocampal texture was superior to volume reduction for the disease prediction. Therefore, we hypothesized that through the earlier involvement of entorhinal cortex and by using texture, it is likely to detect microscopic alterations of the disease before atrophy spreads.

**TABLE 1:** Selected quantitative magnetic resonance imaging (MRI) studies where entorhinal cortex was used for the classification of AD and the prediction of conversion from MCI to AD.

**Author**	**Data type**	**Classification**	**ROI**	**Acc.**	**Se.**	**Sp.**	**Description**
**Classification studies**							
Juottonen et al. (1999)	Volume	NC vs. AD	Hip. Erc.	86% 87%	80% 80%	91% 94%	Both hippocampus and entorhinal cortex had the same discriminative power.
Pennanen et al. (2004)	Volume	NC vs. MCI	Hip. Erc.	60% 66%	57% 65%	62% 70%	Between NC and MCI subjects entorhinal cortex atrophy was more pronounced and provided better classification.
Ryu et al. (2017)	Volume	SMI vs. NC	Hip Erc.	NA	67% 78%	85% 93%	Subjects with SMI had lower Erc. volumes than NC, whereas no differences in Hip volume were seen
**Prediction of conversion from MCI to AD**							
Killiany et al. (2002)	Volume	0 vs. 36 months	Hip. Erc.	NA 84%	NA	NA	Entorhinal cortex differentiated MCI subjects that developed AD, whereas hippocampus did not
deToledo-Morrell et al. (2004)	Volume	0 vs. 36 months	Hip. Erc.	NA 93%	NA	NA	Entorhinal cortex provided better predictive accuracy from hippocampus
Devanand et al. (2007)	Volume	0 vs. 36 months	Hip. Erc.	79% 80%	61% 63%	NA	Entorhinal cortex had more atrophy rates than hippocampus for MCIc
Bakkour et al. (2009)	Thickness	0 vs. 36 months	Cortex	NA	83%	65%	Entorhinal cortex volume may be a better predictor in people with MCI rather than hippocampal volume
Eskildsen et al. (2013)	Thickness	0 vs. 36 months	Cortex	67%–76%	NA	NA	Longitudinal measures in MCI subjects showed that entorhinal cortex was affected first, followed by hippocampus

*SMI, subjective memory impairment; NC, normal controls; MCI, mild cognitive impairment; MCIc, mild cognitive impairment converter; AD, Alzheimer’s disease; ROI, region of interest; Acc., accuracy; Se, sensitivity; Sp, specificity.*

Since we wanted to evaluate the classification and prediction value of MRI, other related to AD features such as CSF Tau, Aβ biomarkers, or ApoE genotyping were not included in the analysis, as they are not generally available in population samples. However, age and gender as main risk factors for developing AD were included.

To the best of our knowledge, this is the first study that used texture analysis on the entorhinal cortex. The main objective in this study was to determine whether MRI entorhinal cortex texture features could detect early cognitive decline in MCI and AD subjects. In addition, we compared entorhinal cortex results to the gold standard method, hippocampal volume, to evaluate which method could provide the best results. We emphasize here that the goal of our research was to investigate the usefulness of entorhinal cortex texture in AD assessment. Then, through a follow-up period of 18 months, we did a comparison between volume and texture measures to evaluate the hypothesis that texture changes may precede the development of atrophy. Finally, we evaluated if entorhinal cortex texture features can be used in the prediction of conversion from MCI to AD.

## Materials and Methods

### The Alzheimer’s Disease Neuroimaging Initiative

Data were acquired from the Alzheimer’s Disease Neuroimaging Initiative (ADNI).^1^ The ADNI was launched in 2003 by the National Institute on Aging, the National Institute of Biomedical Imaging and Bioengineering, the Food and Drug Administration, private pharmaceutical companies, and non-profit organizations as a public–private partnership. The goal of the ADNI study is to determine biological biomarkers of AD through neuroimaging, genetics, neuropsychological tests, and other measures in order to develop new treatments and monitor their effectiveness, and lessen the time of clinical trials.

### Subjects

All subjects selected for this study were from standardized data collections (see http://adni.loni.usc.edu/methods/mri-analysis/adni-standardized-data/) and specifically from the ADNI-1 Complete 2 and 3 year 1.5 Tesla datasets. All data acquired as part of this study are publicly available.^2^ Enrolled subjects were all between 55 and 90 years of age, and each subject was willing, able to perform all test procedures described in the protocol, and had a study partner who is able to provide an independent evaluation of functioning. Overall, 455 subjects were included in the study: 153 NC (73 males and 80 females), 141 MCI (95 males and 46 females), 77 MCI subjects that converted to AD (MCIc; 43 males and 34 females), and 84 AD (40 males and 44 females).

### Cognitive Measures

All subjects underwent through clinical and cognitive assessment at the time of baseline scan to determine their diagnosis. Inclusion criteria for NC were MMSE scores between 24 and 30; CDR of zero; absence of depression, MCI, and dementia. Inclusion criteria for MCI were MMSE scores between 24 and 30; CDR of 0.5; objective memory loss, measured by education adjusted scores on Wechsler Memory Scale Logical Memory II (Elwood, 1991), absence of significant levels of impairment in other cognitive domains, and absence of dementia. Inclusion criteria for AD were MMSE scores between 20 and 26; CDR of 0.5 or 1.0; National Institute of Neurological and Communicative Disorders and Stroke and the Alzheimer’s Disease and Related Disorders Association (NINCDS/ADRDA) criteria for probable AD (McKhann et al., 1984; Dubois et al., 2007). Definitive autopsy-based diagnosis of AD was not possible, and detailed description of inclusion/exclusion criteria can be found in the ADNI protocol.^3^

### MRI Data

All the subjects had a standardized protocol on 1.5-T MRI units from Siemens Medical Solutions and General Electric Healthcare. MR protocols included high-resolution (typically 1.25 × 1.25 × 1.25 mm^3^ voxels) T1-weighted volumetric 3D sagittal magnetization prepared rapid gradient-echo (MPRAGE) scans. MRI data acquisition techniques were standardized across different sites according to the ADNI protocol.^4^

### Segmentation Algorithm and Volumetry

ROI segmentation was performed using the Freesurfer image analysis suite (Massachusetts General Hospital, Boston, MA, United States), which is documented and freely available for download online.^5^ The Freesurfer pipeline conforms the MRI scans to an isotropic voxel size of 1 mm^3^, and the MRI intensity was normalized using the automated N3 algorithm (Sled et al., 1998) followed by skull stripping and neck removal. Details of these have been discussed in previous publications (Fischl et al., 2002, 2004). In brief, this multistep pipeline includes motion correction, automated Talairach transformation, first normalization of voxel intensities, removal of the skull, linear volumetric registration, intensity normalization, non-linear volumetric registration, volumetric labeling, second normalization of voxel intensities, and white matter segmentation. Output includes segmentation of subcortical structures, extraction of cortical surfaces, cortical thickness estimation, spatial normalization onto the FreeSurfer surface template (FsAverage), and parcellation of cortical regions. Hippocampal and entorhinal cortex volumes were computed using Freesurfer segmentations given that this is an established method. Cy-Tera supercomputer of the Cyprus Institute was used to run FreeSurfer.

### Texture Analysis

Texture features were calculated using KNIME Analytics platform (Berthold et al., 2008). The following Haralick texture features (Haralick et al., 1973) were computed: angular second moment (ASM), contrast, correlation, variance, sum average, sum variance, entropy, and cluster shade, and their average in four directions (0°, 45°, 90°, 135°) was used.

### Statistical Analysis

Demographic data along with cognitive tests, texture and volume features of subjects at baseline scans were compared with one-way ANOVA to determine statistical differences between the groups (NC, MCI, MCIc, AD). Then, *post-hoc* tests using the Bonferroni correction were applied to determine if there were significant differences in texture features between the groups. There were no outliers in the data, as assessed by inspection of a boxplot.

Texture features and volume were combined as predictor variables in a logistic regression model in order to investigate the potential of combined value of the two MRI biomarkers. Backward elimination methods were used to select the most suitable variables. Apart from texture and volume, we included age and gender as covariates. Through receiving operating characteristic (ROC) curves, we determined the performance of the various variables, and their ability to discriminate NC from MCI and AD subjects, as well as to classify the conversion status. The resulting area under the curve (AUC) was used to determine the capability for diagnosis. The significance of an AUC was determined using DeLong, Delong, and Clarke–Pearson’s test (DeLong et al., 1988).

Then, through a repeated measures ANOVA, we compared entorhinal cortex and hippocampal volume changes with texture changes within 18 months and evaluated if there were significant texture changes during follow-up period. Data were checked for outliers and normal distribution, as assessed by boxplot and Shapiro–Wilk test (*p* > 0.05). When sphericity was violated, as assessed by Mauchly’s test, the Greenhouse–Geisser correction was applied. Then, *post-hoc* tests using the Bonferroni correction was used to compare the volume and texture changes.

To evaluate the prognostic power of our model, we also used AUC curves on MCI and MCIc subjects. Specifically, the MCI group was randomly divided into both a training set (∼70% of the participants) and a trial set (∼30%) of the participants. This was iterated five times to provide five unique training and test groups. The training sets were used to fit two binary logistic regression models: the first model included entorhinal cortex volume, MMSE scores, age, and gender as covariates, and the second model had the same features, plus entorhinal cortex texture to determine if the addition of texture-based metrics could improve the accuracy. For a more robust prediction model, the colinearity between the predictor variables was evaluated, and only those for which the colinearity was acceptable were included in the final model. The estimated logistic regression model was then applied to the validation cohorts.

Statistical analysis was performed with IBM SPSS Statistics Version 24 (IBM Corp. Released 2011; IBM SPSS Statistics for Windows, Version 20.0. Armonk, NY: IBM Corp.) or MedCal Version 19 (MedCalc Software bvba, Ostend, Belgium).^6^ The significance level of all statistical tests was set at *P* < 0.05.

## Results

Baseline demographics including gender, age, and MMSE scores are shown in Table 2. All baseline variables (except the age) were significantly different between the four groups based on one-way ANOVA. Estimations of hippocampal and entorhinal cortex volumes are in cubic millimeter (left and right averaged). As expected, AD patients had smaller volumes than MCI subjects, and both had smaller volumes than NC subjects.

**TABLE 2:** Baseline demographics and hippocampal and entorhinal cortex volume.

**Variables at baseline (mean ± SD)**	**Diagnosis group**				***P-*value**
	**NC (*n* = 194)**	**MCI (*n* = 200)**	**MCI_c (*n* = 84)**	**AD (*n* = 130)**	
Sex (M/F)	96/98	127/73	49/35	60/70	0.003
Age	76.17 (5.20)	74.74 (7.18)	74.88 (7.30)	76.01 (7.35)	0.111
MMSE score	29 (1.0)	27 (1.82)	26 (1.84)	23 (2.16)	<0.001
CDR	0.0 (0.00)	0.50 (0.04)	0.50 (0.00)	0.75 (0.25)	<0.001
Entorhinal cortex volume (mm^3^)	1,930 (284)	17,191,723 (384)	154,410 (338)	1,417 (348)	<0.001
Hippocampal volume (mm^3^)	3,539 (413)	3,243 (461)	2,941 (461)	2,892 (474)	<0.001

*NC, normal controls; MCI, mild cognitive impairment; MCIc, mild cognitive impairment converter; AD, Alzheimer’s disease; MMSE, mini-mental status examination; CDR, clinical dementia rating; SD, standard deviation.*

### Between-Group Differences

A one-way ANOVA was conducted to determine if there were significant texture features between the groups for baseline scans (Table 3). For NC vs. AD and NC vs. MCI groups, entorhinal cortex revealed statistically significant differences in more features compared to hippocampus. Specifically, hippocampus did not show any significant changes for NC vs. MCI group, apart for volume. However, hippocampal texture revealed statistical significant differences in more features between MCI vs. MCIc group. Between MCI vs. AD, both structures had similar results with entorhinal cortex showing statistically significant differences in the texture feature contrast, correlation, sum variance, and entropy, whereas hippocampus for the texture feature ASM, sum average, and entropy.

**TABLE 3:** Mean differences at baseline scans for entorhinal cortex and hippocampus.

	**Mean difference (SE)**							
	**Entorhinal cortex**				**Hippocampus**			
**Group**	**NC vs. AD**	**NC vs. MCI**	**MCI vs. MCIc**	**MCI vs. AD**	**NC vs. AD**	**NC vs. MCI**	**MCI vs. MCIc**	**MCI vs. AD**
**Texture features**								
ASM	−0.017*(0.005)	−0.01(0.004)	−0.007(0.006)	−0.006(0.005)	−0.013*(0.004)	−0.008(0.004)	−0.023*(0.005)	−0.023*(0.005)
Contrast	−024.3*(2.60)	−7.73*(2.27)	−7.36(2.93)	−16.60*(2.60)	−2.0(2.36)	−3.61(2.11)	−4.62(2.70)	−5.60(2.32)
Correlation	−0.051*(0.005)	−0.017*(0.004)	−0.013(0.005)	−0.034*(0.005)	−0.0004(0.005)	−0.0004(0.005)	−0.016(0.007)	−0.001(0.005)
Variance	−0.201(1.14)	−1.11(1.01)	−0.780(1.30)	−0.910(1.14)	−2.40(2.80)	−4.90(2.46)	−13.0*(2.20)	−7.30(2.77)
Sum average	−1.44*(0.365)	−0.590(0.324)	−0.821(0.414)	−0.853(0.361)	−1.64*(0.560)	−0.823(0.498)	2.40*(0.640)	−2.47*(0.556)
Sum variance	−27.70*(4.15)	−12.28*(3.68)	−2.18(4.80)	−15.41*(4.14)	−6.30(10.0)	−15.20(8.80)	−41.0*(11.3)	−21.5(9.80)
Entropy	−0.137*(0.023)	−0.058*(0.020)	−0.060(0.026)	−0.080*(0.023)	−0.103*(0.028)	−0.034(0.024)	−0.113*(0.030)	−0.138*(0.026)
Cluster shade	−471(350)	−260(312)	−118*(414)	−730(342)	−1,009(1,038)	−2,373(927)	−4,600*(1182)	−3,383(1,026)
**Volumetric features**								
Volume	−513*(39.1)	−211*(34.8)	−174*(45.0)	−301*(38.7)	−646*(52.4)	−295*(42.7)	−302*(60.0)	−351*(51)

SE, standard error; ASM, angular second moment; NC, normal controls; MCI, mild cognitive impairment; MCIc, mild cognitive impairment converter; AD, Alzheimer’s disease. *The mean difference is significant at the 0.05 level.

### Texture Differences Between Groups – Classification

To determine the classification between the groups, a binary logistic regression model was calculated for each individual variable, and using ROC curves, we determined their AUC. The combination model included raw single MRI variable scores as well as age and gender as covariates. In most of the cases, all eight texture features revealed significant differences between groups (see Tables 4–6). Then, all the variables were combined together, and the backward elimination method selected the more important predictor variables.

**TABLE 4:** Entorhinal cortex texture and volume in classifying NC vs. AD.

**NC vs. AD**	**ROC analysis AUC**	**95% CI**	***P-*value**
**Entorhinal cortex**			
**Texture features**			
ASM	0.592	0.529–0.656	0.005
Contrast	0.794	0.743–0.845	<0.001
Correlation	0.824	0.776–0.872	<0.001
Variance	0.524	0.458–0.590	0.475
Sum average	0.620	0.555–0.681	<0.001
Sum variance	0.713	0.653–0.770	<0.001
Entropy	0.685	0.625–0.744	<0.001
Cluster shade	0.540	0.475–0.604	0.238
**Volume and thickness**			
Erc. volume	0.888	0.847–0.925	<0.001
Erc. thickness	0.809	0.755–0.863	<0.001
Features combination			
Texture (ASM, correlation, variance, sum average, and cluster shade)	0.872	0.828–0.916	<0.001
Texture + Erc. volume	0.914	0.879–0.950	<0.001
**Hippocampus**			
Hippocampal volume	0.869	0.827–0.912	<0.001

*NC, Normal Controls; AD, Alzheimer’s disease; ROC, receiver operating characteristic; AUC, area under curve, CI, confidence interval; ASM, angular second moment; Erc, entorhinal cortex.*

**TABLE 5:** Entorhinal cortex texture and volume in classifying NC vs. MCI.

**NC vs. MCI**	**ROC analysis AUC**	**95% CI**	***P*-value**
**Entorhinal cortex**			
**Texture features**			
ASM	0.618	0.563–0.674	<0.001
Contrast	0.664	0.611–0.718	<0.001
Correlation	0.671	0.617–0.725	<0.001
Variance	0.604	0.548–0.660	<0.001
Sum average	0.618	0.562–0.674	<0.001
Sum Variance	0.641	0.586–0.695	<0.001
Entropy	0.632	0.577–0.687	<0.001
Cluster shade	0.608	0.551–0.666	<0.001
**Volumetric and thickness**			
Erc. volume	0.735	0.686–0.784	<0.001
Erc. thickness	0.659	0.604–0.713	<0.001
**Features combination**			
Texture (ASM, correlation, variance, sum average, and cluster shade)	0.710	0.656–0.762	<0.001
Texture and Erc. volume	0.740	0.689–0.791	<0.001
**Hippocampus**			
Hippocampal volume	0.762	0.715–0.809	<0.001

*NC, normal controls; MCI, mild cognitive impairment; ROC, receiver operating characteristic; AUC, area under curve, CI, confidence interval; ASM, angular second moment; Erc, entorhinal cortex.*

**TABLE 6:** Entorhinal cortex texture and volume in classifying MCI vs. MCIc.

**MCI vs. MCIc**	**ROC analysis AUC**	**95% CI**	***P-*value**
**Entorhinal cortex**			
**Texture features**			
ASM	0.565	0.494–0.637	0.85
Contrast	0.583	0.510–0.657	0.028
Correlation	0.580	0.505–0.654	0.038
Variance	0.531	0.458–0.604	0.037
Sum average	0.591	0.520–0.662	0.036
Sum variance	0.527	0.451–0.603	0.475
Entropy	0.593	0.522–0.662	0.014
Cluster shade	0.696	0.632–0.759	0.032
**Volume and thickness**			
Erc. volume	0.642	0.573–0.711	<0.001
Erc. thickness	0.670	0.603–0.737	<0.001
**Features combination**			
Texture (ASM, correlation, variance, sum average, and cluster shade)	0.730	0.665–0.795	<0.001
Texture and Erc. volume	0.756	0.692–0.820	<0.001
**Hippocampus**			
Hippocampal volume	0.685	0.617–0.753	<0.001

*MCIc, mild cognitive impairment converter; ROC, receiver operating characteristic; AUC, area under curve, CI, confidence interval; ASM, angular second moment; Erc, entorhinal cortex.*

For NC vs. AD group, the AUC for entorhinal cortex texture values ranged from 0.540 to 0.824 (Table 4). When texture features were combined into a single classification model, the AUC reached 0.872, which was similar to hippocampal volume (AUC 0.869) and entorhinal cortex volume (AUC 0.888). When entorhinal cortex texture and volume were combined, the AUC reached 0.914.

Between NC and MCI subjects, features combination showed a lower AUC (0.710) compared to entorhinal and hippocampal volume (Table 5). The entorhinal cortex texture and volume combination raised the AUC to 0.740.

Between MCI and MCIc subjects, features combination provided a higher AUC (0.730), compared to entorhinal cortex and hippocampal volume (Table 6). The entorhinal cortex texture and volume combination raised the AUC to 0.756.

Between MCI and AD subjects, features combination showed a higher AUC of 0.764 compared to entorhinal cortex and hippocampal volume (Table 7). The entorhinal cortex texture and volume combination raised the AUC to 0.780.

**TABLE 7:** Entorhinal cortex texture and volume in classifying MCI vs. AD.

**MCI vs. AD**	**ROC analysis AUC**	**95% CI**	***P*-value**
**Entorhinal cortex**			
**Texture features**			
ASM	0.627	0.565–0.690	<0.001
Contrast	0.704	0.646–0.763	<0.001
Correlation	0.725	0.668–0.783	<0.001
Variance	0.624	0.560–0.688	<0.001
Sum average	0.649	0.587–0.711	<0.001
Sum variance	0.658	0.596–0.720	<0.001
Entropy	0.656	0.594–0.718	<0.001
Cluster shade	0.645	0.583–0.706	<0.001
**Volume and thickness**			
Erc. volume	0.726	0.670–0.781	<0.001
Erc. thickness	0.702	0.642–0.762	<0.001
**Features combination**			
Texture (ASM, correlation, variance, sum average, and cluster shade)	0.764	0.710–0.818	<0.001
Texture + Erc. volume	0.780	0.728–0.833	<0.001
**Hippocampus**			
Hippocampal volume	0.711	0.652–0.771	<0.001

*MCI, mild cognitive impairment; AD, Alzheimer’s disease; ROC, receiver operating characteristic; AUC, area under curve, CI, confidence interval; ASM, angular second moment; Erc, entorhinal cortex.*

### Measures Between Different MRI Scan Intervals

A one-way repeated measures ANOVA was conducted to determine whether there were statistically significant differences in entorhinal cortex (texture and volume) over the 18-month observation (baseline, 6, 12, and 18 months). For comparison, hippocampal volume was also included in this analysis. At each time point, a diagnosis was made based on the NINCDS-ADRDA Alzheimer’s Criteria to identify conversion of MCI to probable AD and vice versa, and only MCI and MCIc subjects were included in this part of the analysis. Specifically, longitudinal data of 141 MCI and 77 MCIc subjects were included in this analysis. The means and standard deviations for volume are presented in Table 8 and for texture in Table 9. We reported the *F*-statistic from the repeated measures ANOVA test as *F*(df_time_, df_error_) = *F*-value, *P* = *P*-value.

**TABLE 8:** Statistically significant difference in entorhinal cortex and hippocampal volume over an 18-month intervention.

**Within-subjects effects**	**Entorhinal cortex volume Mean (SD) mm^3^**		**Hippocampal volume Mean (SD) mm^3^**	
	**MCI**	**MCIc**	**MCI**	**MCIc**
Baseline	1,733 (390)	1,504 (293)	3,263 (483)	2,906 (439)
6 months	1,712 (389)	1,449 (278)	3,213 (487)	2,860 (423)
12 months	1,693 (393)	1,424 (269)	3,168 (496)	2,819 (432)
18 months	1,657 (411)	1,384 (269)	3,129 (487)	2,781 (436)
*F*-ratio (Time)	*F*(2.837, 383) = 45.62, *P* < 0.0005	*F*(3, 186) = 45.06, *P* < 0.0005	*F*(2.748, 376) = 41.8, *P* < 0.0005	*F*(3, 195) = 21.74, *P* < 0.0005

*MCI, mild cognitive impairment; MCIc, mild cognitive impairment converters; SD, standard deviation.*

**TABLE 9:** Statistically significant difference in entorhinal cortex texture over an 18-month intervention.

	**Entorhinal cortex texture features – mean (SD)**							
	**ASM**	**Contrast**	**Correlation**	**Sum variance**	**Variance**	**Sum variance**	**Entropy**	**Cluster shade**
**MCI stable**								
Baseline	0.226 (0.047)	220 (22.38)	0.498 (0.043)	659.4 (38.0)	220 (9.49)	659 (38.0)	2.92 (0.216)	1,838 (3,218)
6 months	0.228 (0.046)	223 (20.95)	0.495 (0.041)	659.2 (38.5)	221 (9.90)	659 (38.5)	2.90 (0.221)	1,198 (,2986)
12 months	0.230 (0.047)	223 (19.34)	0.494 (0.043)	656.0 (38.1)	220 (9.50)	655 (41.9)	2.89 (0.212)	1,276 (3,086)
18 months	0.234 (0.047)	222 (24.71)	0.493 (0.049)	654.0 (38.1)	219 (10.7)	654 (44.3)	2.89 (0.212)	1,300 (2,989)
*F*-ratio (Time)	*F*(3, 417) = 2.45 *P* = 0.620	*F*(3, 384) = 1.05 *P* = 0.370	*F*(3, 375) = 1.14 *P* = 0.332	*F*(3, 411) = 2.17 *P* = 0.09	*F*(3, 411) = 1.44 *P* = 0.230	*F*(3, 411) = 2.17 *P* = 0.091	*F*(3, 414) = 2.65 *P* = 0.048	*F*(3, 396) = 2.49 *P* = 0.060
**MCI converters**								
Baseline	0.230 (0.041)	230 (26.6)	0.480 (0.046)	655.4 (35.0)	221 (10.5)	655 (35.0)	2.87 (0.179)	-296.7 (1,860)
6 months	0.237 (0.039)	238 (27.4)	0.468 (0.043)	650.0 (40.5)	222 (11.4)	650 (40.5)	2.82 (0.185)	-388.4 (2,363)
12 months	0.235 (0.040)	237 (28.5)	0.468 (0.047)	647.4 (42.0)	222 (12.0)	637 (42.0)	2.82 (0.186)	-385.0 (2,394)
18 months	0.243 (0.043)	238 (28.8)	0.459 (0.047)	637.8 (44.3)	219 (12.7)	647 (44.3)	2.79 (0.156)	-557.1 (2,032)
*F*-ratio (Time)	*F*(2.72, 179.5) = 3.86 *P* = 0.013	*F*(3, 201) = 5.80 *P* < 0.0005	*F*(3, 192) = 6.82 *P* < 0.0005	*F*(3, 201) = 7.45 *P* < 0.0005	*F*(3, 198) = 3.55 *P* < 0.0005	*F*(3, 201) = 7.45 *P* < 0.0005	*F*(3, 204) = 7.30 *P* < 0.0005	*F*(3, 162) = 0.23 *P* = 0.874

*MCI, mild cognitive impairment; ASM, angular second moment; SD, standard deviation.*

For entorhinal cortex volume in both MCI and MCIc subjects, there was a significant effect for time [*F*(2.837, 383.0) = 45.62, *P* < 0.0005] and [*F*(3, 186) = 45.06, *P* < 0.0005], respectively. Furthermore, the mean difference was statistically significant at the 0.05 level between all-time points for both MCI and MCIc subjects with the exception of 12–18 time points for MCIc subjects. *Post-hoc* tests using the Bonferroni correction showed that entorhinal cortex volume in the MCI subjects was reduced by an average of 20 ± 6.9 mm^3^ 6 months after the baseline scan, then by an additional 19 ± 6.0 mm^3^ between 6 and 12 months’ time and 36 ± 6.1 mm^3^ between 12 and 18 months’ time. As expected, the entorhinal cortex degeneration was more pronounced in the MCIc subjects. Their entorhinal cortex volume was reduced by an average of 55 ± 10.5 mm^3^ 6 months after the baseline scan, then by an additional 25 ± 9.5 mm^3^ between 6 and 12 months’ time and 40 ± 10.3 mm^3^ between 12 and 18 months’ time.

For hippocampal volume in both MCI and MCIc subjects, there was significant effect for time [*F*(2.748, 376.4) = 41.8, *P* < 0.0005] and [*F*(3, 195) = 21.74, *P* < 0.0005] respectively. Furthermore, the mean difference was statistically significant at the 0.05 level between all-time points for both MCI and MCIc subjects with the exception of 12–18 time points for MCIc subjects. Interestingly, hippocampal volume reduction in the MCIc subjects was similar to MCI stable subjects. Specifically, *post-hoc* tests using the Bonferroni correction revealed that hippocampal volume in the MCI subjects was reduced by an average of 49 ± 12.6 mm^3^ 6 months after the baseline scan, then by an additional 45 ± 11.5 mm^3^ between 6 and 12 months’ time and 38 ± 11.3 mm^3^ between 12 and 18 months’ time. A similar pattern was seen in MCIc subjects as well as hippocampal volume reduction was 45 ± 15.8 mm^3^ after 6 months from the baseline scan, and then reduced by an additional 40 ± 14.0 mm^3^ between 6 and 12 months’ time and an additional 38 ± 16.6 mm^3^ between 12 and 18 months’ time.

Remarkably, repeated measures ANOVA in the entorhinal cortex texture features of MCIc subjects revealed that there was significant effect for time for all features (except for cluster shade), whereas in stable MCI subjects, there was significant effect for time only for sum variance and entropy.

### Prediction of Conversion to AD Within 18 Months

To evaluate entorhinal cortex texture in the prediction of conversion from MCI to AD, all the MCI subjects were divided into two categories: the MCI subjects who remained stable and did not convert to AD within 18 months (*n* = 200) vs. the MCIc subjects who converted to AD within 18 months (*n* = 84). First, we run a prediction model, which included entorhinal cortex volume, MMSE scores, and gender with age as covariates. Then, a second model was run where entorhinal cortex texture features (contrast and cluster shade) were included as well, to evaluate if texture metrics could improve accuracy. The selected variables were also evaluated for colinearity between them, and their degree of correlation was acceptable.

Then, the MCI group was divided into a training set (∼70) and a trial set (∼30%). We randomly generated five of these sets, with each training set having a total of *n* = 133 MCI and *n* = 55 MCIc, whereas the trial set had total of *n* = 67 MCI and *n* = 29 MCIc. Independent sample *t*-test and chi-square analysis showed no statistical difference between the baseline demographics in the training and trial sets in each iteration. For each of the two models, five binary logistic regression models were determined, corresponding to one for each training set (Table 10). The model including texture performed better and achieved AUCs of 0.795, 0.725, 0.745, 0.786, and 0.750, respectively. Then, the logistic regression coefficients from the final model developed from the training cohorts were applied to the validation cohorts, and AUCs of 0.780, 0.780, 0.790, 0.735, and 0.735 were seen.

**TABLE 10:** Area under curve in five trials of randomly splitting training (70%) and trial data (30%).

	**Entorhinal cortex volume**						**Entorhinal cortex volume and texture**					
**Trial**	**Training cohort**			**Validation cohort**			**Training cohort**			**Validation cohort**		
	**ROC analysis AUC**	**(95% CI)**	***P-*value**	**ROC analysis AUC**	**(95% CI)**	***P-*value**	**ROC analysis AUC**	**(95% CI)**	***P-*value**	**ROC analysis AUC**	**(95% CI)**	***P-*value**
1	0.760	0.690–0.830	0.000	0.700	0.583–0.814	0.000	0.795	0.728–0.862	0.000	0.780	0.662–0.898	0.000
2	0.673	0.595–0.751	0.001	0.688	0.573–0.804	0.005	0.725	0.649–0.801	0.000	0.780	0.674–0.886	0.001
3	0.663	0.580–0.746	0.04	0.658	0.538–0.778	0.005	0.745	0.770–0.820	0.000	0.790	0.680–0.903	0.001
4	0.662	0.582–0.742	0.000	0.635	0.500–0.772	0.005	0.786	0.712–0.860	0.000	0.735	0.621–0.848	0.001
5	0.647	0.565–0.730	0.001	0.709	0.591–0.827	0.003	0.751	0.675–0.827	0.000	0.735	0.627–0.843	0.001

*ROC, receiver operating characteristic; AUC, area under curve, CI, confidence interval.*

## Discussion

The main objective of this study was to evaluate entorhinal cortex texture as a new biomarker of AD from T1-weighted MR images. To the best of our knowledge, this is the first study that used texture analysis on the entorhinal cortex for the assessment of AD. Thus, our results are not directly compared to the same method and ROI previously used in other AD studies, but mainly to hippocampal volume, which represents the most frequently used method in the assessment of AD. In the analysis, apart from entorhinal cortex texture features, we calculated also its volume, and we combined them in a binary logistic regression model, which included age and gender as covariates.

For entorhinal cortex, one way-ANOVA showed that contrast, correlation, and volume were the features that showed statistical significant differences between all groups and, for hippocampus, sum average, cluster shade, and volume (see Table 3). For the NC vs. MCI group, one-way ANOVA showed that were statistically significant differences in more features for the entorhinal cortex compared to the hippocampus, whereas the hippocampus showed significant differences in more features between MCI vs. MCIc group. Perhaps, these differences are correlated with the fact that entorhinal cortex is the region affected first by the disease (Gómez-Isla et al., 1997; Juottonen et al., 1999; Galton et al., 2001; Killiany et al., 2002; Busatto et al., 2003; deToledo-Morrell et al., 2004; Tapiola et al., 2008), whereas the hippocampus is involved in a later stage.

In the literature, the entorhinal cortex and hippocampus have shown a significant role in the assessment of AD (Leandrou et al., 2018). Similarly, in the present study, results of the ROC curve analysis showed that for the entorhinal cortex, there were significant differences between NC subjects and AD patients. Specifically, there were significant texture changes in six texture features (apart from variance and cluster shade), and their combination provided an AUC of 0.872 (*P* < 0.001) for the discrimination between NC and AD subjects (Table 4). This was similar to entorhinal cortex or hippocampal volume, which showed AUC of 0.888 (*P* < 0.001) and 0.869 (*P* < 0.001), respectively. When entorhinal cortex texture features and volume were combined into the same model, the diagnostic result was improved, showing an AUC of 0.914 (*P* < 0.001).

Compared to a study that used hippocampal texture such as from Zhang et al. (2012), their classification accuracy reached 96.4%. However, their dataset included severely affected AD subjects (MMSE 5.53 ± 4.47 compared to 23 ± 1.9 for the ADNI data in the present study). Compared to the study of Sørensen et al. (2015), which also used the ADNI dataset, their hippocampal texture achieved an AUC of 0.912 in discriminating NC from AD. On the other hand, the study of Luk et al. (2018) used texture features on the whole brain, and the combination of hippocampal texture features and volume provides an AUC of 0.924, which was close to our combined model.

Compared to other ADNI volumetric studies where hippocampus was used, NC subjects were classified from AD patients with AUC levels of 0.750–0.887 (Mueller et al., 2010) and 0.810–0.895 when hippocampal subfields only were used (Khan et al., 2015). This is comparable to our hippocampal volume results (AUC 0.869), which is close to entorhinal cortex texture (AUC 0.888). In other studies (Juottonen et al., 1999; Colliot et al., 2008), where both hippocampal and entorhinal cortex volume were used, the classification accuracy ranged between 84 and 86%. In the study of Pennanen et al. (2004), the combination of hippocampal and entorhinal cortex volume provided an accuracy of 91%.

Between NC vs. MCI subjects, the combination of entorhinal cortex texture features in the logistic regression model provided an AUC of 0.710 and their combination with entorhinal cortex volume raised the AUC to 0.740 (Table 5). This is comparable to the AUC (0.764) in the study of Sørensen et al. (2015) where hippocampal texture was used. In the study of Hwang et al. (2016) where voxel-based 3DT1W was used on the whole brain, their AUC ranged between 0.682 and 0.713, which was close to our single ROI method. The study by Simoes et al. (2012) used whole brain texture maps reaching a classification accuracy of 87% (Se. 85%, Sp. 95%); however, their analysis was based on 3-Tesla (T) images. Compared to other studies (Pennanen et al., 2004; Colliot et al., 2008) that used hippocampal volume for the discrimination between NC vs. MCI, a classification accuracy close to 66% was achieved.

Between MCI and MCIc subjects, the combination of entorhinal cortex texture features and volume provided an AUC of 0.756, whereas entorhinal cortex or hippocampal volume provided lower AUCs of 0.642 and 0.685, respectively. Our result for this group was similar to the study of Chincarini et al. (2011) where texture features were extracted from defined volumes of interest, mainly from the MTL, and through random forest classifiers, they achieved an AUC of 0.740. These findings suggest that the entorhinal cortex texture changes precede neuronal atrophy of the hippocampus, which is consistent with the most widely used staging scheme proposed by Braak and Braak (1991). Specifically, Stage I is associated with NFT deposition in the entorhinal–perirhinal cortex, and in Stage II, the NFTs become more prominent, and the entorhinal cortex is eventually involved. In Stage III, the entorhinal cortex is fully involved, whereas between Stages III and IV, NFTs appear in the hippocampus. Eventually, in Stages V–VI, apart from the MTL, NFTs are also widely distributed in the isocortex.

Between MCI and AD subjects, the combination of entorhinal cortex features showed better diagnostic capability (AUC of 0.764) compared to entorhinal cortex and hippocampal volume (AUCs of 0.726 and 0.711, respectively). The combination of entorhinal cortex texture and volume raised the AUC to 0.780 (Table 7). For this group, other studies (Pennanen et al., 2004; Colliot et al., 2008; Ferrarini et al., 2009) achieved a classification accuracy between 80 and 82% using volumetric or shape characteristics of the hippocampus and entorhinal cortex.

In the one-way repeated measures ANOVA, the entorhinal cortex volume reduction was more pronounced in the MCIc subjects, whereas hippocampal volume atrophy rate was similar in both MCI and MCI subjects. Similar finding was seen in the study of Devanand et al. (2007) where it was shown that entorhinal cortex had more severe atrophy rates, compared to hippocampus, in MCIc subjects. Regarding entorhinal cortex texture features (Table 9), the one-way repeated measures ANOVA showed significant effect for time (for all texture features) in MCIc subjects, whereas in MCI stable subjects, there was no statistically significant difference (apart from entropy). Perhaps, this finding indicates that through entorhinal cortex texture features, we could identify that MCI subjects in the future could develop the disease.

Furthermore, we determined whether entorhinal cortex texture could be used to predict conversion of MCI to AD within 18 months. For the discrimination of stable MCI from MCIc subjects, our prediction model including entorhinal cortex features, volume, MMSE scores, age, and gender performed better rather than volume alone and demonstrated an average AUC of 0.760 in the training cohort and an AUC of 0.764 in the validation cohort. In this study, the combination of texture and volume features improved the prediction of conversion from MCI to AD, and this was also the finding as well by two recent studies by Gao et al. (2018) and Luk et al. (2018). Compared to other studies that followed their subjects for the same time period, such as from Chupin et al. (2009) and Cuingnet et al. (2011), hippocampal volume was used, and the classification accuracy between MCI and MCIc was 67 and 64%, respectively. Sørensen et al. (2015), compared hippocampal volumetry and texture in the differentiation between stable MCIs and MCI converters within 24 months, and AUCs of 0.670 and 0.740, respectively, were achieved. In the study from Misra et al. (2009) where a VBM method on the whole brain was used to consider the conversion within 12 months, an accuracy of 81.5% was obtained. In a recent study by Lee et al. (2020) texture analysis was also used for the prediction of the disease in subjects from the ADNI database. In their analysis texture of the hippocampus, precuneus and posterior cingulate cortex were included, and their model ranged between AUCs of 0.79–0.82, whereas our one structure only analysis ranged between 0.735 and 0.790.

There are some limitations in the present study. First, the ADNI cohort cannot be generalized to the normal population given that the patient recruitment was targeted toward clinical trials in patients with AD. The baseline demographics of these sample patients do not fit with the actual demographics of the broader population. For example, female/male ratio is poor with almost twice as many males as females especially for MCI subjects. Furthermore, ADNI study does not provide post-mortem pathological confirmation of the clinical status. Therefore, the stable MCI subjects we selected for the present study although did not progress to AD within the followed-up period, they might have developed the disease or other types of dementia in a later stage. Therefore, as in any AD study involving *in vivo* data, the diagnosis of the disease remains probable. Thus, MRI patterns of neurodegeneration found in studies like the present may have uncertainties.

To our knowledge, this is the first study that runs texture analysis on the entorhinal cortex for the assessment of AD to identify texture changes in the classification of MCI and AD subjects. Furthermore, it evaluated if entorhinal cortex texture can be used in the prediction of the disease development, and according to our results, in many cases, entorhinal cortex texture changes provided better results compared to the hippocampal atrophy, which remains the most frequent method in the assessment of AD. This suggests that the deposition of NFTs in the area of entorhinal cortex may precede the development of atrophy in the hippocampus.

## Data Availability Statement

The datasets generated for this study are available on request to the corresponding author.

## Ethics Statement

The studies involving human participants were reviewed and approved by the ADNI Data Sharing and Publications Committee. The patients/participants provided their written informed consent to participate in this study.

## Author Contributions

SL contributed to the design and implementation of the research and performed the numerical calculations for the suggested experiment. DL and IM contributed to the statistical analysis. CP supervised the project and contributed to the manuscript preparation and revision. PK read and approved the final submitted version. All authors contributed to the article and approved the submitted version.

## Conflict of Interest

The authors declare that the research was conducted in the absence of any commercial or financial relationships that could be construed as a potential conflict of interest.

## Footnotes

1 http://adni.loni.usc.edu/

2 http://adni.loni.usc.edu/data-samples/

3 http://adni.loni.usc.edu/methods/documents/

4 http://adni.loni.usc.edu/methods/documents/mri-protocols/

5 http://surfer.nmr.mgh.harvard.edu/

6 https://www.medcalc.org; 2019
