# Corrigendum: Assessment of Alzheimer's Disease Based on Texture Analysis of the Entorhinal Cortex

**DOI:** 10.3389/fnagi.2020.596070

**Published:** 2020-10-22

**Authors:** Stephanos Leandrou, Demetris Lamnisos, Ioannis Mamais, Panicos A. Kyriacou, Constantinos S. Pattichis

**Affiliations:** ^1^School of Science, European University Cyprus, Nicosia, Cyprus; ^2^School of Mathematical Sciences, Computer Science and Engineering, City, University of London, London, United Kingdom; ^3^Department of Computer Science, University of Cyprus, Nicosia, Cyprus; ^4^Research Centre on Interactive Media, Smart Systems and Emerging Technologies (RISE CoE), Nicosia, Cyprus

**Keywords:** Alzheimer's disease, mild cognitive impairment, entorhinal cortex, magnetic resonance imaging, texture

In the original article, there was a mistake in Table 6 as published. The first column title was “NC vs. MCI” and it should be “MCI vs. MCIc.” The corrected Table 6 appears below.

**TABLE 6:** Entorhinal cortex texture and volume in classifying MCI vs. MCIc.

**MCI vs. MCIc**	**ROC analysis AUC**	**95% CI**	***P-*value**
**Entorhinal cortex**			
**Texture features**			
ASM	0.565	0.494–0.637	0.85
Contrast	0.583	0.510–0.657	0.028
Corelation	0.580	0.505–0.654	0.038
Variance	0.531	0.458–0.604	0.037
Sum average	0.591	0.520–0.662	0.036
Sum variance	0.527	0.451–0.603	0.475
Entropy	0.593	0.522–0.662	0.014
Cluster shade	0.696	0.632–0.759	0.032
**Volume and thickness**			
Erc. volume	0.642	0.573–0.711	<0.001
Erc. thickness	0.670	0.603–0.737	<0.001
**Features combination**			
Texture (ASM, correlation, variance, sum average, and cluster shade)	0.730	0.665–0.795	<0.001
Texture & Erc. volume	0.756	0.692–0.820	<0.001
**Hippocampus**			
Hippocampal volume	0.685	0.617–0.753	<0.001

*MCIc, mild cognitive impairment converter; ROC, receiver operating characteristic; AUC, area under curve; CI, confidence interval; ASM, angular second moment; Erc, entorhinal cortex*.

The authors apologize for this error and state that this does not change the scientific conclusions of the article in any way. The original article has been updated.

